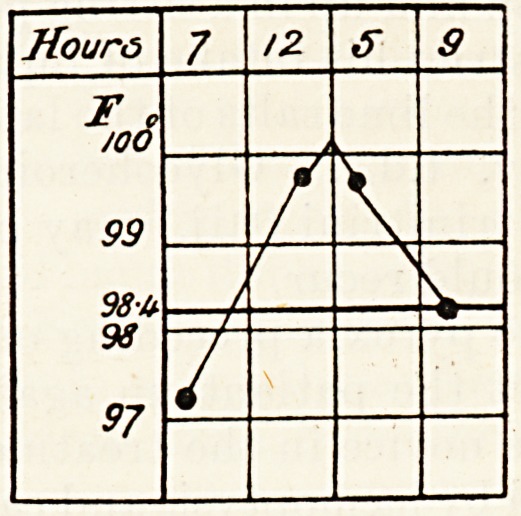# Treatment of Pulmonary Phthisis

**Published:** 1907-12-07

**Authors:** W. C. Rivers

**Affiliations:** Late Assistant Physician Vale of Clwyd and Crossley Sanatoria.


### THE GENERAL PRACTITIONER'S COLUMN.

[Contributions to this Column are invited, and if accepted will be paid for.]

### TREATMENT OF PULMONARY PHTHISIS.

By W. C. RIVERS, L.R.C.P., M.R.C.S., D.P.H., Late Assistant Physician Vale of Clwyd and Crossley Sanatoria.

EVEN to-day, when sanatoria have multiplied so much, there is still a large number of consumptives the treatment of whom falls to the general practitioner. Most of these, of course, belong to the poorer classes. If they seek admission to a public institution the advanced stage of their disease often renders them ineligible. But there are other classes of consumptive patients, in whose case the general practitioner may find opportunity for carefully detailed and often successful treatment. Such are, first of all, children of both sexes. Parents are often unwilling to part with them and fear the effect of separation on the child's spirits. A sanatorium, besides, however carefully conducted, is perhaps not a very suitable place for a child. Secondly, cases of consumption are still often—very unwisely—sent to live in some particular locality, and their treatment naturally falls to the practitioners they find there. Then one meets with morbid individuals who will not face the many changes involved in institution life. A few others leave sanatoria of their own accord and many more are sent away on various grounds. Nearly all of these seek advice sooner or later of the general practitioner.

From the nature of the case—it is a simple matter to open windows and to discard dust-attracting fabrics such as curtains—the subject of ventilation may be briefly dismissed. It should be said, however, that the principle of the "through current" must be adopted. A current of fresh air should, if possible, be freely and appreciably moving from a considerable aperture at one side of the patient's apartment to a similar one opposite, preferably impinging gently upon the patient in its course. It will be easily seen, then, that this is better carried out in a hut than in a room. The exceptions are cases of laryngeal tuberculosis of pre-existing chronic bronchitis. For the vast majority of cases, the more open air and the less artificial heat the better. Indeed, with thorough ventilation consumptives do much better in winter than in summer. The lower temperature seems to have a tonic effect.

Exercise and thermometry are of the highest importance. The great principle is to treat all departures from the optimum range of temperature by increased rest. It is not generally known that under open-air conditions the daily range of oral temperature of the consumptive whose disease is quiescent is somewhat as follows:—

Rectal temperature differs slightly from oral; if the patient is having exercise the two middle readings show its effect, if taken immediately afterwards. The following chart shows a model rectal temperature, the patient taking walking exercise morning and afternoon:—

The first temperature is taken immediately on waking; the two next at the hours stated, immediately after coming in from exercise, if the patient is ordered any; the last one ten minutes after getting into bed. The patient takes his own temperature and keeps his own chart.

If the patient be not on exercise, the rectal temperature will, except in the early morning, average about half a degree higher than the oral. Now, of course, there may be extraneous causes of pyrexia, but transgressions of these various upward limits means generally some activity of the disease, as do also pronounced flushing and dilated pupils towards the evening. Irregularity of temperature must therefore be met by rest in bed. At one large private sanatorium every patient spends his first fortnight in bed. This may be a little arbitrary, but it is certain that the general adoption of this plan would do more good than harm. If the pyrexia is obstinate, so must the physician be. Months in bed may be necessary: irresolution and vacillation are in this respect disastrous. Apyrexia once secured, exercise may be carefully begun. It must consist of nothing else but walking; the pace must not exceed two and a half miles an hour, and the patient is to sit down and rest at intervals. The distance, of course, is very gradually increased up to eight or ten miles a day in suitable cases. Always, twice a day, the rectal temperature is taken immediately on coming in. If it exceeds 100.4° F. the exercise must be lessened. Instructions may be left to this effect. Oral temperature is not so useful, as it will not guide one as to the suitability of exercise. High winds are bad for laryngeal cases, otherwise weather is immaterial.

As for diet, this must of course vary with the amount of the patient's exercise or pyrexia. Very high temperatures require mainly liquid diet. With slight pyrexia there is the danger that a patient on full diet and no exercise may put on weight too rapidly, and run the risk of hæmoptysis and dyspepsia. Purgatives are here useful, and individual judgment must be used. As a general thing, two pounds a week for adults is a useful rate of gain. Three full meals a day are required, at stated hours, and each accompanied by (for adults and adolescents) a pint of hot milk. In the general run of cases, one should aim at getting the patient about half a stone heavier than he has ever been. Before dinner and supper the patient must recline on a couch for an hour and rest. He should be in bed by 9 p.m. Nothing at all is to be given between meals; alcohol is quite unnecessary.

With respect to complications, one thinks first of ordinary capillary hæmoptysis. Reassure the patient and prop him up in a sitting position. Order a purge and restrict his intake of liquids, especially milk. Replace the lime salts of the latter by calcium chloride gr. xv. t.d.s. Glycoheroin will relieve cough, and liq. trinitrini (ᵐij.) may be added if the hæmorrhage should recur.

If there be no pyrexia preceding or following the hæmoptysis, get the patient up again soon. It is the mark of the novice in the treatment of phthisis to be frightened by hæmoptysis and careless of slight pyrexia. Recumbency, too, probably encourages pulmonary hæmorrhage.

For laryngeal cases two months' complete silence —the patient should learn the deaf and dumb alphabet—combined with the treatment above indicated will often work a vast improvement. This is, indeed, acknowledged to be by far the most important remedial measure in laryngeal tuberculosis.

Here, then, given rather dogmatically, is the outline of the systematic treatment of consumption. It is, naturally, a routine of life which is much easier to carry out in a special institution than elsewhere. Nevertheless, if the physician can secure his patient's intelligent interest and co-operation, the result should in favourable cases be gratifying and the monthly physical examinations show steady improvement.

If the patient can afford the necessary time and money—and this is pre-supposed in all that has been said—the mode of life described should always be continued for a year.

## Figures and Tables

**Figure f1:**
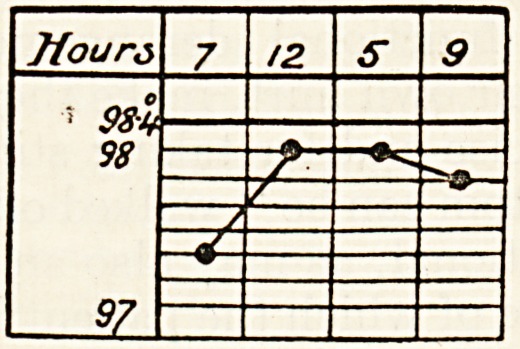


**Figure f2:**